# Case Report: Endovascular embolization under DSA for multivessel supply mixed pulmonary sequestration in young patients

**DOI:** 10.3389/fcvm.2024.1518853

**Published:** 2025-01-20

**Authors:** Hong Li, Qinhong Li, Xing Zhang, Feng Li, Zhongjian Su, Lili Deng, Chuxiong Gong

**Affiliations:** ^1^ Department of Cardiology, Kunming Children’s Hospital, Kunming, Yunnan, China; ^2^ Department of Cardiology, The First Affiliated Hospital of Kunming Medical University, Kunming, Yunnan, China; ^3^ Integrated Pediatrics, Kunming Children’s Hospital, Kunming, Yunnan, China

**Keywords:** pulmonary sequestration, interventional therapy, DSA, aorta, children's diseases

## Abstract

Pulmonary sequestration (PS) is a rare congenital lung developmental anomaly characterized by abnormal lung tissue that receives its blood supply from the aorta. Among the various types, mixed PSrepresents a distinct subtype. Currently, there is a lack of definitive reports regarding the specific management of this condition in infants and young children. We report a case of a 1-year-old patient with mixed PS treated with endovascular occlusion under digital subtraction angiography (DSA). Given the patient's young age and the complexity of the vascular malformation, we opted for endovascular intervention rather than surgery. Utilizing the Seldinger technique, we accessed the right femoral artery and vein for angiography, confirming the abnormal vessels supplying the pulmonary cyst. Subsequently, we placed vascular embolization devices to achieve closure. Postoperatively, the patient did not experience any significant complications. Follow-up revealed substantial absorption of the PS, disappearance of the abnormal vessels originating from the descending aorta, and no recurrence of respiratory infections. This case highlights the efficacy of endovascular intervention in young patients with complex vascular mixed PS.

## 1 Introduction

PS is a rare congenital lung malformation characterized by abnormal lung tissue supplied by aberrant systemic arteries, which lacks direct connection to the bronchial tree. Based on anatomical features, PS is classified into intralobar and extralobar types (1–3). However, some patients exhibit significant anatomical characteristics of both types, resulting in mixed PS. With the increasing prevalence of prenatal cardiac ultrasound and advancements in technology, the detection rate of PS has gradually risen, leading to younger ages at diagnosis (4, 5). Treatment options for PS primarily include traditional thoracotomy, minimally invasive thoracoscopic surgery, and endovascular embolization under digital subtraction angiography (DSA). DSA embolization, particularly in infants and young children, offers advantages such as minimal trauma and rapid recovery. This article reports a case of a 1-year-old patient with mixed PS supplied by multiple vessels, who achieved favorable outcomes following endovascular embolization under DSA.

## 2 Manuscript formatting

### 2.1 Case presentation

The patient was a 1-year-old boy. He presented with a 10-day history of recurrent cough accompanied by fever. Over the past 10 days, he experienced recurrent cough with sputum production and intermittent fever, with a peak temperature of 39.2°C. He received intravenous treatment at a local clinic without improvement (specific medication details were unclear) and subsequently visited our outpatient department. A CT scan (3D reconstruction of the heart and airways) revealed four tortuous and malformed arterial vessels branching from the descending aorta entering the dorsal segment of the left lower lung. A relatively large malformed venous vessel returned to the right atrial orifice of the superior vena cava in the left lower lung. There was also a small amount of fluid in the left pleural cavity. Additionally, cardiac ultrasound indicated a malformed arterial vessel branching leftward from the descending aorta, accompanied by mitral and tricuspid regurgitation. Therefore, we initially suspected PS and admitted the patient for treatment. Upon admission, the physical examination showed slight cyanosis of the lips, a positive/negative retraction sign, and a transcutaneous oxygen saturation of 90% without supplemental oxygen. The respiratory rate was 36 breaths per minute, with coarse breath sounds in both lungs and fixed fine crackles in the left lower lung. The heart rate was 140 beats per minute, with a regular rhythm and no significant heart murmurs detected. The patient had a tendency for upper respiratory tract infections. Inquiring about the medical history, the mother reported that a prenatal check at four months of pregnancy indicated fetal pulmonary vascular abnormalities (the specifics were unclear, and the report had been lost). There was no family history of hereditary pulmonary vascular malformations. The patient had experienced three recurrent pulmonary infections in the past six months, all of which improved after intravenous treatment at a local clinic (specific intravenous details were unclear), and his activity endurance was slightly weaker than that of peers. Considering the patient's young age, the significant trauma associated with open chest and thoracoscopic surgeries, the still immature development of the child's lungs, the difficulty in lesion dissection, and the complexity of the four abnormal vessels with diverse return pathways, we opted for vascular interventional embolization following angiography under DSA.

**Figure 1:**
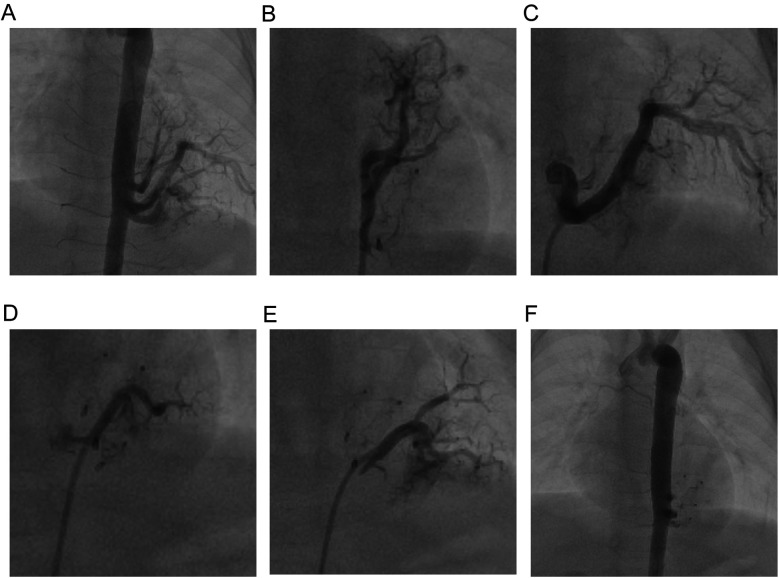
Abnormal branch angiography of the descending aorta. **(A)** Angiography of the four abnormal vessels before surgery. **(B–E)** Individual angiograms of the four abnormal vessels from head to foot. **(F)** Angiography of the four abnormal vessels after surgery.

We used the Seldinger technique to puncture the right femoral artery and vein, and we inserted a TERUMO 5Fr arterial sheath and a 6Fr venous sheath, while administering heparin (100 U/kg) to the patient. Then, with the assistance of a Philips Allura Xper FD20 DSA (The total radiation dose during the surgical procedure was 82mSv.), we performed a standard descending aorta angiography using a Cordis 5Fr Pigtail catheter and a selective vascular angiography using a TERUMO Cobra C2 catheter (The contrast agent is Iopamidol, manufactured by Bayer Pharmaceuticals, with a dosage of 60 ml and 37.4 g). We found four malformed arterial vessels originating from the left posterior wall of the descending aorta at the level of the 7th–8th thoracic vertebrae supplying a pulmonary cyst (Figure 1A). A small portion of blood flow returned to the left atrium through collateral vessels into the pulmonary veins, while most flowed through the malformed vascular network, ascending along the abnormal venous vessels beside the spine to converge at the junction of the superior vena cava and the right atrium (Figure 1A). We then sequentially used a Beijing Huayi Shengjie 0.032 × 260 mm super smooth guidewire to navigate from the descending aorta into the malformed vessel in the direction of distal blood flow. A COOK multipurpose (MPA) 5Fr catheter was guided along the super smooth guidewire into the malformed vessel, and the guidewire was withdrawn to perform angiography of the four malformed arteries (Figures 1A–E). At this point, we noticed that the vessels from the aorta to the pulmonary cyst were relatively large. Using a spring coil for embolization could easily lead to thrombus entering the pulmonary veins or the right atrium early on, and later it might cause an aneurysm at the origin of the abnormal vessels from the aorta to the pulmonary cyst. Therefore, we chose to use vascular plugs for embolization (which was more reasonable to position). Subsequently, we used a Cordis 0.035 Inch–150 cm 502–521 stiff guidewire to navigate along the 5Fr-MPA catheter into the malformed vessel in the direction of distal blood flow, establishing a pathway from the right femoral artery to the malformed vessel. After withdrawing the MPA catheter, we inserted a Shanghai shape memory 4Fr occluder delivery device along the stiff guidewire and delivered a total of four 8 mm Shanghai shape memory vascular plugs to the distal end of each malformed vessel. We then positioned the distal umbrella of the vascular plugs at an appropriate location at the distal end of the malformed vessels, with the waist fixed within the malformed vessel and the proximal umbrella secured near the opening of the descending aorta. Angiography of the malformed vessels showed no significant shunt after releasing the vascular plugs. Angiography of the descending aorta indicated complete occlusion of the four malformed vessels with no shunting observed, and the proximal end of the vascular plugs was located within the opening of the malformed arteries in the descending aorta (Figure 1F).

**Figure 2:**
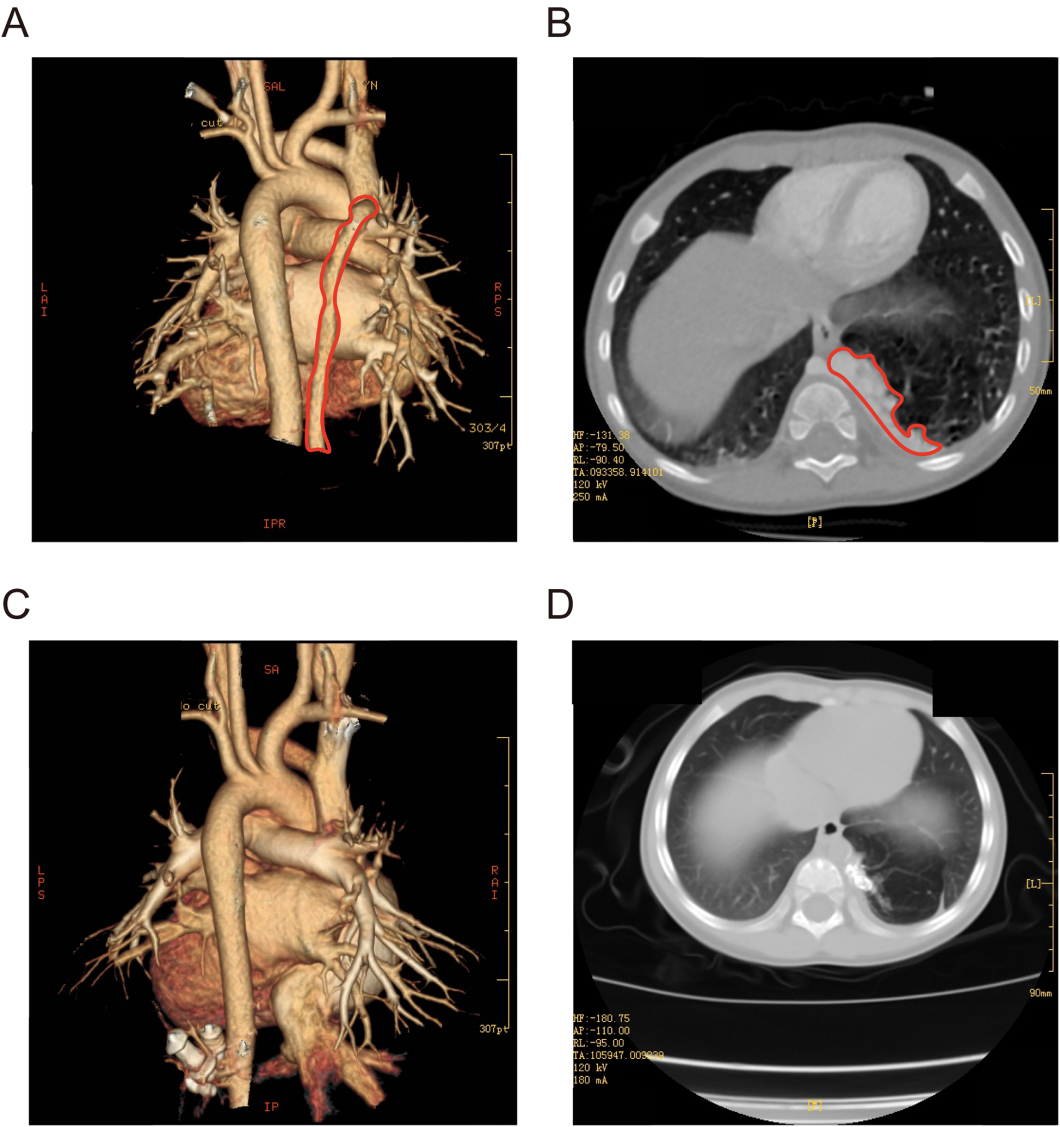
**(A,B)** Three-dimensional CT of the chest before surgery (marked in red for PS). **(C,D)** Three-dimensional CT of the chest 12 months after surgery.

The child underwent vascular interventional embolization under DSA and experienced fever for 3 days, which resolved with simple anti-inflammatory and anti-infective treatment. No other complications such as respiratory distress, cyanosis, chest pain, oliguria, or edema occurred. Postoperative echocardiography indicated no abnormal blood flow in the aorta, and there was a reduction in mitral and tricuspid regurgitation. Follow-up urine analysis and renal function tests were within normal limits. During a follow-up six months post-surgery, echocardiography did not reveal any residual shunting. Comparing the preoperative CT (Figures 2A,B) with the follow-up CT at 12 months post-surgery, most of the abnormal lung tissue had been absorbed and regressed, with no significant vascular configuration in the non-regressed portions (Figures 2C,D). The child did not experience any upper respiratory infections or pneumonia in the 12 months following the surgery, gained 3 kg in weight, and showed improved activity endurance. The family expressed satisfaction with the treatment outcome.

## 3 Discussion

Currently, reports on mixed PS are relatively scarce, especially with no clear cases documented in young children. The vascular anatomy of mixed PS is more complex, which can easily lead to right-to-left shunting of arterial blood, resulting in hypoxia. In our case, we observed four abnormal vessels originating from the descending aorta: one returned to the superior vena cava, two returned to the pulmonary veins, and the last one returned to both the superior vena cava and the pulmonary veins. The complex system of return vessels increased the volume load of the returning blood, potentially leading to increased right heart load, elevated pulmonary circulation blood flow, and resulting in recurrent pulmonary infections, pulmonary hypertension, and heart failure. Furthermore, the incomplete visceral pleura increased blood flow exchange with normal lung tissue, leading to increased blood flow in the alveolar vascular network, which raises the risk of rupture and hemorrhage (5).

Liu, C et al. reviewed the records of 42 patients with PS treated between September 2005 and May 2012 at a single institution. Among these patients, 18 underwent video-assisted thoracoscopic surgery (VATS) and 24 underwent posterolateral thoracotomy. In the VATS group, one patient converted to open surgery due to arterial injury. One patient experienced left lower pulmonary vein injury, and another had an anomalous artery injury. The conclusion stated that VATS for PS was feasible but should be performed by experienced surgeons who are aware of the potential risk of severe vascular injury (6). Li, Q et al. reviewed data from 110 adult patients who underwent thoracoscopic surgery or open surgery for lobar sequestration at their institution between January 2000 and December 2015. They identified 42 cases of video-assisted thoracoscopic surgery (VATS) and 68 cases of open surgery. The study compared perioperative outcomes between VATS and open surgery. The results indicated that thoracoscopic surgery resulted in less intraoperative bleeding and shorter postoperative hospital stays compared to open surgery (7). Xu Feng et al. reported a case of a 34-year-old male, a non-smoker, who suddenly experienced massive hemoptysis. CT angiography suggested intralobular PS. They performed selective embolization of the feeding vessels of the sequestrated lung tissue via a femoral artery approach. Post-embolization angiography revealed that the blood supply to the sequestrated area had been completely removed. Seven days after the embolization, the patient's hemoptysis symptoms resolved, and he had no complications at discharge. During a one-year follow-up, no recurrent infections or hemoptysis were detected (8). L Mariné et al. reported a case of a 23-year-old female who was hospitalized due to a history of recurrent respiratory infections and three episodes of hemoptysis. Computed tomography and magnetic resonance imaging confirmed the diagnosis of PS. The patient underwent endovascular treatment, which included the use of an Amplatzer occlusion device and spring coil embolization of the feeding vessels. Subsequent computed tomography angiography confirmed complete occlusion of the sequestrated region. Compared to traditional surgery, endovascular treatment of PS with selective embolization of the feeding arteries is an attractive minimally invasive option and may be associated with fewer related complications (9).

The treatment options for mixed-type PS faced significant challenges. In infants, the unique anatomical structure and smaller surgical field made lung resection surgery highly difficult. Additionally, the physiological instability and limited lung reserve in infants further increased the perioperative risks. During surgery, adhesiolysis could raise the risk of accidental injury to the feeding arteries. Therefore, compared to traditional open thoracic surgery and thoracoscopic surgery, endovascular embolization treatment resulted in less trauma, less bleeding, faster recovery, and lower rates of intraoperative adverse events and postoperative complications. Furthermore, the immature lung tissue in infants exhibited better self-differentiation and repair potential, suggesting that the removal of avascular cysts might yield better long-term outcomes than surgical resection.

It is noteworthy that approximately 58.6% of PS cases were misdiagnosed, with 36.19% misdiagnosed as lung cysts and 21.04% as lung cancer. In the case of the child in this study, the history of recurrent pulmonary infections prompted a DSA examination based on chest CT, leading to a definitive diagnosis and subsequent endovascular intervention. The follow-up showed that the child recovered well, with no further recurrent lung infections. One year postoperatively, the child's weight increased by 3 kg, and exercise tolerance significantly improved. Thus, endovascular treatment allowed for preoperative angiography to accurately depict the anatomical structure of the systemic circulation, aiding in confirming the diagnosis of PS and effectively avoiding missed or incorrect diagnoses.

The primary difficulty in this case lay in placing multiple occluders within a confined operational space. Moreover, due to the shunting from four vessels, contrast agent could appear on both sides of the closed vessels before completing the embolization of the four abnormal vessels. Determining the residual shunt after closing the first three vessels proved challenging. Therefore, we utilized preoperative angiography to understand the shape of the vessels, which assisted in designing the order of closure and the embolization plan.

## 4 Conclusions

In summary, we reported a case of endovascular embolization treatment for a 1-year-old child with mixed-type PS under DSA. Despite the child's young age, multiple large abnormal vessels, and complex venous return, the endovascular treatment led to a favorable prognosis with no significant complications. The cardiopulmonary function and nutritional status improved significantly, and family satisfaction was high. This case provided a theoretical reference for the diagnosis and treatment of mixed-type PS in infants and offered case support for the endovascular embolization treatment of multivascular mixed-type PS.

## Data Availability

The original contributions presented in the study are included in the article/Supplementary Material, further inquiries can be directed to the corresponding author.
